# Peripheral TERT- positive leukocytes as a biomarker of the local and systemic immune failure in early-stage lung adenocarcinoma progression

**DOI:** 10.1186/s43556-025-00383-3

**Published:** 2025-12-19

**Authors:** Qi Zhang, Xiaoli Zhang, Guoliang Li, Ligong Yuan, Duo Wan, Peipei Xie, Shujun Cheng, Yu Zhang, Kaitai Zhang, Yousheng Mao, Wen Zhang

**Affiliations:** 1 Department of Gastroenterology, Beijing Friendship Hospital, Capital Medical University, State Key Laboratory for Digestive Health, National Clinical Research Center for Digestive Diseases, Beijing, 100032, China; 2 State Key Laboratory of Molecular Oncology, Department of Etiology and Carcinogenesis, National Cancer Center/National Clinical Research Center for Cancer/Cancer Hospital, Chinese Academy of Medical Sciences and Peking Union Medical College, Beijing, 100021 China; 3 Department of Radiation Oncology, The Affiliated Hospital of Qingdao University, Qingdao, 266000, China; 4 Department of Thoracic Surgery, the First Affiliated Hospital of USTC, Division of Life Sciences and Medicine, University of Science and Technology of China, Hefei, 230001 China; 5 Department of Immunology, National Cancer Center/National Clinical Research Center for Cancer/Cancer Hospital, Chinese Academy of Medical Sciences and Peking Union Medical College, Beijing, 100021 China; 6 Department of Thoracic Surgery, National Cancer Center/National Clinical Research Center for Cancer/Cancer Hospital, Chinese Academy of Medical Sciences and Peking Union Medical College, Beijing, 100021 China

**Keywords:** Lung adenocarcinoma, Invasive adenocarcinoma, Adenocarcinoma in situ, Minimally invasive adenocarcinoma, Immunosuppression

## Abstract

The prognosis of early-stage lung adenocarcinoma (LUAD) worsens significantly during the progression from adenocarcinoma in situ/minimally invasive adenocarcinoma (AIS/MIA) to invasive adenocarcinoma (IAC), emphasizing the importance of understanding the molecular mechanisms underlying this transition for effective clinical intervention. This study analyzed data from 990 eligible early-stage LUAD patients meeting the following inclusion criteria: written informed consent, absence of neoadjuvant therapy, planned surgical resection, postoperative pathological confirmation of LUAD, and an Eastern Cooperative Oncology Group (ECOG) performance status score of 0 or 1. Analysis of a retrospective cohort of 601 stage I LUAD patients demonstrated a significant correlation between the extent of tumor invasion and 5-year survival rates. Transcriptomic profiling revealed a progressively immunosuppressive tumor microenvironment (TME) during the AIS/MIA-to-IAC transition, characterized by dysfunctional immune cells and diminished local anti-tumor immunity. Notably, peripheral blood analysis from 389 patients showed a significant decrease in telomerase reverse transcriptase-positive (TERT +) leukocytes with tumor progression, a change detectable earlier than circulating tumor cells (CTCs). Two-year follow-up data corroborated these findings. This study elucidates key biological events in early-stage LUAD progression, revealing that the tumor remodels both local and systemic immunity to facilitate its advancement. The reduction in peripheral TERT + leukocytes emerges as a potential non-invasive biomarker for the early detection of malignant progression, supporting the rationale for immune-based interventions at earlier disease stages.

**Supplementary Information:**

The online version contains supplementary material available at 10.1186/s43556-025-00383-3.

## Introduction

Lung adenocarcinoma (LUAD) represents more than 75% of lung cancer cases and progresses stepwise from the preinvasive to the invasive stage, as defined by international multidisciplinary guidelines [1]. This spectrum includes atypical adenomatous hyperplasia (AAH), adenocarcinoma in situ (AIS), minimally invasive adenocarcinoma (MIA), and invasive adenocarcinoma (IAC), with AAH, AIS, and MIA collectively categorized as preinvasive LUAD (preIAC) [2–4]. Advancements in screening and diagnostic technologies, such as low-dose computed tomography (LDCT) and liquid biopsy (e.g., ctDNA, cfDNA, CTC), have significantly improved early detection and staging of LUAD [5]. Importantly, even within stage I disease, prognosis varies considerably among pathological subtypes: patients with preIAC have nearly 100% 5-year progression-free survival (PFS), whereas patients with stage I IAC have a cumulative 5-year recurrence rate of 17.9% [6, 7].

The prognosis of LUAD declines sharply from preIAC to IAC, suggesting the critical importance of understanding the molecular events driving this transition. Several studies have investigated genomic, proteomic, and immunological alterations during lung cancer progression [8, 9]. Genomic changes, such as tumor protein p53 (TP53) mutations, arm-level copy number changes, and increased loss of heterozygosity in human leukocyte antigen (HLA) genes, are implicated in invasion and immune evasion [10]. Proteomic studies have shown that the deletion of chr4q12 is a key event in the AIS/MIA-to-IAC transition. The deletion of chr4q12 downregulates SPATA18 expression, thereby inhibiting mitophagy and promoting cell invasion and proliferation [11]. Analysis of T-cell receptor (TCR) diversity and immunohistochemical staining revealed a shift toward an immunosuppressive state, potentially leading to immune failure [12, 13]. Given that genomic changes are ultimately translated into phenotypic changes through RNA-level alterations, comprehensive transcriptomic studies characterizing the molecular biology of the AIS/MIA-to-IAC transition are needed. Such studies could further our understanding of LUAD pathogenesis and development, potentially revealing promising therapeutic targets.

While studies have described LUAD progression within the primary tumor site, the impact of early-stage LUAD on the systemic environment, particularly the systemic immune status, remains unclear. Given that tumor recurrence and metastasis involve systemic mobilization, a broader perspective on patient-level changes is essential. Aberrant expression of telomerase reverse transcriptase (TERT) is a key mechanism in tumorigenesis [14], and normal TERT expression is crucial for immune cell proliferation and function. Previous research has indicated that hTERT expression is associated with T-cell proliferation and immune responses [15–17], as well as with the expansion of memory B cells [18]. Thus, TERT expression in immune cells may serve as a valuable indicator of systemic immune status, enabling a holistic assessment of immune reserve and responsiveness.

**Fig. 1:**
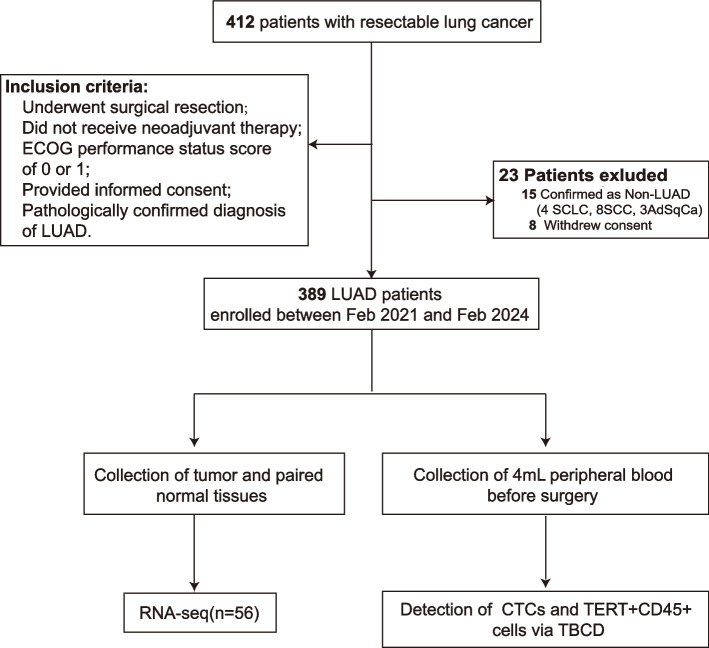
Flowchart of the prospective cohort study

In this study, survival analysis of a retrospective cohort of 601 patients with stage I LUAD confirmed that tumor progression was an independent prognostic factor, even in early disease stages. Consequently, a systematic investigation of early-stage LUAD progression offers two important benefits: revealing initial tumorigenic mechanisms and identifying potential targets for clinical intervention to improve patient outcomes. Therefore, this study focused on local and systemic immuno-tumor interactions accompanying the transition from pre-IAC to IAC (Fig. 1). At the local level, transcriptomic sequencing was performed on tumors and adjacent normal tissues across different progression stages (from AAH to IAC) to examine dynamic changes in the tumor microenvironment. At the systemic level, circulating tumor cells (CTCs) were detected in peripheral blood to assess tumor burden, and the proportion of TERT + leukocytes was analyzed to reflect systemic immune status (Fig. S1). By integrating local transcriptomic features with peripheral blood indicators, this study provides multidimensional insights into the mechanisms of early-stage LUAD progression, offering new avenues for clinical intervention.

## Results

### Stage I LUAD invasion depth was significantly associated with the risk of recurrence and metastasis

**Fig. 2:**
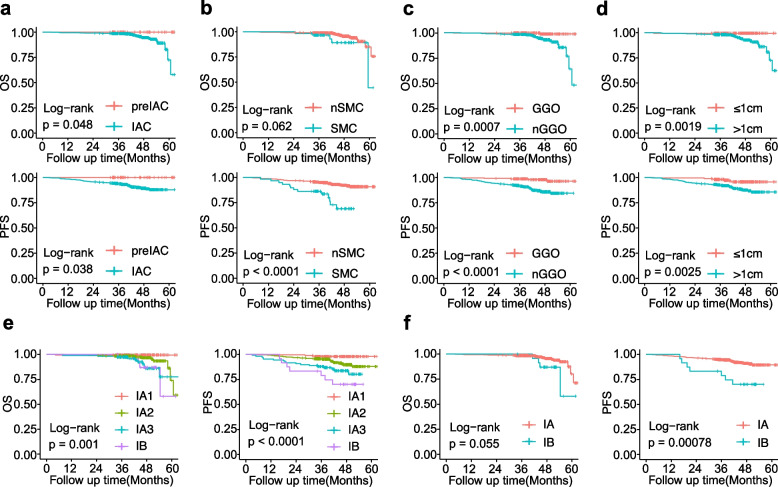
Characteristics of recurrence and metastasis in patients with stage I LUAD. **a-d** Five-year survival analysis of a retrospective cohort of 601 cases. Kaplan–Meier curves for overall survival (OS) and progression-free survival (PFS) in patients with stage I lung adenocarcinoma(LUAD) stratified according to pathological subtype (**a**), histologic characteristics (**b**), imaging characteristics (**c**), and tumor size (**d**). **e–f** Kaplan–Meier curves for OS and PFS in patients with stage I LUAD stratified according to tumor stage. *p*-values were calculated by the log-rank test

To investigate the risk of recurrence and metastasis in early-stage LUAD, a 5-year retrospective analysis of 601 patients with stage I LUAD was conducted (clinical and pathological characteristics are listed in Table 1). The recurrence and metastasis rate was 8.5% (51/601), with metastases observed in the lung, brain, bone, lymph nodes, contralateral pleura, and liver. Critically, metastases occurred exclusively in patients with the invasive subtype of LUAD; patients with the pre-invasive subtype had 100% overall survival (OS) and progression-free survival (PFS). The differences in OS (*p* = 0.048) and PFS (*p* = 0.038) between the two subtypes were statistically significant (Fig. 2a). Histological features (solid and micropapillary components, SMC), imaging characteristics (non-ground glass opacity, nGGO), and tumor size (> 1 cm) were significant factors influencing recurrence and metastasis in early-stage LUAD (Fig. 2b–d). Within Stage I LUAD, substaging had a significant role in survival stratification, with OS (*p* = 0.001) and PFS (*p* < 0.0001) decreasing with advancing substaging (Fig. 2e–f). Cox regression analysis corroborated these findings (Fig. S2). These results indicate that the risk of recurrence and metastasis significantly increases with tumor progression and invasion depth, even in early-stage disease. Therefore, understanding the biological and molecular changes that occur during the transition from the pre-invasive to the invasive stage is crucial for elucidating the mechanisms underlying recurrence and metastasis.

**Table 1:** Clinical and pathological characteristics of the 5-year follow-up retrospective cohort of 601 patients with stage I LUAD

Characteristics	Patients surviving without disease (*n* = 550)	Patients with recurrence and metastasis (*n* = 51)	*p* value
Tumor size (average, cm)	1.42	1.86	0.276
Neurological violation, n (%)	3(0.5)	2(3.9)	0.011
Vessel carcinoma embolus, n (%)	14(2.5)	7(13.7)	< 0.001
Median age (range, years)	57(28–74)	60(44–74)	0.006
Gender, n (%)			< 0.001
Female	371(67.5)	21(41.2)	
Male	179(32.5)	30(58.8)	
Smoking history, n (%)			0.015
Smoker	127(23.1)	24(47.1)	
Nonsmoker	423(76.9)	27(52.9)	
Clinical TNM stage, n (%)			< 0.001
IA1	189(34.4)	4(7.8)	
IA2	253(46.0)	23(45.1)	
IA3	91(16.5)	17(33.3)	
IB	17(3.1)	7(13.7)	
Pathology, n (%)			0.026
AIS	4(0.7)	0	
MIA	45(8.2)	0	
IAC	501(91.1)	51(100)	
Adjuvant therapy, n (%)			< 0.001
Chemotherapy	1(0.2)	5(9.8)	
Radiotherapy	1(0.2)	0	
Chemotherapy + Radiotherapy	0	3(5.8)	
Radiological features, n (%)			< 0.001
Solid	237(43.1)	40(78.4)	
Sub-Solid	97(17.6)	6(11.8)	
GGO	216(39.3)	5(9.8)	
Surgical procedure, n (%)			0.015
Lobectomy	442(80.4)	48(94.1)	
Sublobar resection	108(19.6)	3(5.9)	
Histomorphology, n (%)			< 0.001
SMC	79(14.4)	27(52.9)	
nSMC	471(85.6)	24(47.1)	
Tumor location, n (%)			0.887
LUL	142(25.8)	14(27.5)	
LLL	83(15.2)	7(13.7)	
RUL	187(34.0)	17(33.3)	
RML	37(6.6)	2(3.9)	
RLL	101(18.4)	11(21.6)	

*n (%)*:the number of patients within each category(corresponding percentage of that category), *AIS* adenocarcinoma in situ, *MIA* minimally invasive adenocarcinoma, *IAC* invasive subtype of lung adenocarcinoma, *GGO* ground glass opacity, *SMC* solid and micropapillary components, *nSMC* non-solid and micropapillary components, *LUL* left upper lobe, *LLL* left lower lobe, *RUL* right upper lobe, *RML* right middle lobe, *RLL* right lower lobe

### Transcriptome landscape shifts upon early-stage LUAD invasion

**Fig. 3:**
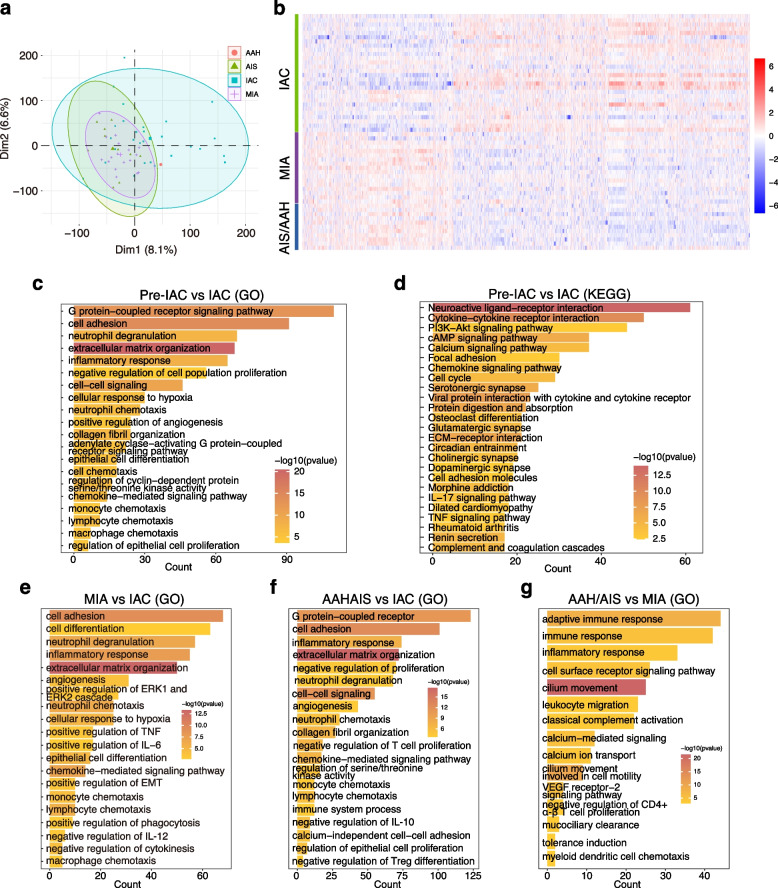
Transcriptome landscape of different subtypes of early-stage LUAD. **a** Principal component analysis (PCA) plot of different pathological stages of progression from atypical adenomatous hyperplasia (AAH)/ adenocarcinoma in situ (AIS) to and invasive adenocarcinoma (IAC). **b** Heatmap of differential gene analysis for AAH/AIS, minimally invasive adenocarcinoma (MIA) and IAC. **c**-**d** Gene Ontology (GO) and Kyoto Encyclopedia of Genes and Genomes (KEGG) enrichment analysis of differentially expressed genes before and after tumor invasion (preIAC vs IAC). **e**-**g** GO enrichment analysis of differential genes between stages of pathological progression, including MIA vs IAC (**e**), AAH/AIS vs IAC (**f**), AAH/AIS vs MIA (**g**)

To elucidate the molecular dynamics during the progression of early-stage LUAD, transcriptome sequencing was performed on 56 tumor tissues representing stages from AAH, AIS, and MIA to IAC, along with their adjacent normal tissues. Clinical information for these samples is provided in Table 2. Principal component analysis (PCA) revealed distinct clustering of pre-IAC and IAC samples. AAH/AIS and MIA exhibited extensive overlap, suggesting limited transcriptomic alterations before invasion. Conversely, IAC samples were broadly distributed, partially overlapping with pre-IAC samples, indicating increased transcriptomic heterogeneity after invasion. These findings suggest that the expression profiles of early-stage LUAD differ significantly from those of preinvasive LUAD (Fig. 3a and S3a). Differential gene expression analysis confirmed pronounced expression profile differences between pre-IAC and IAC samples, with distinct clustering (Fig. 3b). Differences between the AAH/AIS and MIA samples were less pronounced (Fig. S3b).

**Table 2:** Patient characteristics of the early-stage LUAD prospective cohort (*n* = 389) and the RNA-sequencing cohort (*n* = 56)

Characteristics	Prospective cohort (*n* = 389)	RNA-seq cohort (*n* = 56)	*p* value
Tumor size (average, cm)	1.744	1.63	0.649
Vessel carcinoma embolus, n (%)	39(10.0)	6(10.7)	1.000
Median age (years, range)	59(26–85)	60(36–78)	0.597
Gender, n (%)			0.544
Male	131(33.7)	16(28.6)	
Female	258(66.3)	40(71.4)	
Smoking history, n (%)			< 0.001
Smoker	319(82.0)	11(19.6)	
Nonsmoker	70(18.0)	45(80.4)	
Clinical TNM stage, n (%)			0.003
0-I	339(87.1)	48(85.7)	
II	19(4.9)	8(14.3)	
III	31(8.0)		
Pathology, n (%)			0.017
AAH	1(0.3)	1(1.8)	
AIS	41(10.5)	10(17.8)	
MIA	77(19.8)	17(30.4)	
IAC	270(69.4)	28(50.0)	
Radiological features, n (%)			0.557
Solid	105(27.0)	19(33.9)	
Sub-Solid	138(35.5)	18(32.2)	
GGO	146(37.5)	19(33.9)	
Surgical procedure, n (%)			0.505
Lobectomy	138(35.3)	23(41.1)	
Sublobar resection	251(64.7)	33(58.9)	
Surgical procedure, n (%)			
Lobectomy	138(35.3)	23(41.1)	
Sublobar resection	251(64.7)	33(58.9)	
Histomorphology, n (%)			0.624
SMC	135(34.7)	17(30.4)	
nSMC	254(65.3)	39(69.6)	
Tumor location, n (%)			0.716
LUL	112(28.8)	14(25.0)	
LLL	48(12.3)	8(14.3)	
RUL	122(31.4)	20(35.7)	
RML	23(5.9)	5(8.9)	
RLL	84(21.6)	9(16.1)	

*n(%)*:the number of patients within each category(corresponding percentage of that category), *AIS* adenocarcinoma in situ, *MIA* minimally invasive adenocarcinoma, *IAC* invasive subtype of lung adenocarcinoma, *GGO* ground glass opacity, *SMC* solid and micropapillary components, *nSMC* non-solid and micropapillary components, *LUL* left upper lobe, *LLL* left lower lobe, *RUL* right upper lobe, *RML* right middle lobe, *RLL* right lower lobe

GO and KEGG enrichment analyses of subtype-specific differential genes indicated that compared with IAC samples, pre-IAC samples were enriched in pathways related to cell adhesion, angiogenesis, and immune cell chemotaxis (Fig. 3c–g and S3c–f). AAH/AIS and MIA did not significantly affect cell adhesion (Fig. 3e), suggesting that decreased cell adhesion primarily occurs during invasion. However, angiogenesis-related signals, such as VEGF receptor expression, were upregulated as early as the AAH/AIS stage (Fig. 3e–f). Alterations in the immune response were evident throughout tumor progression: earlier stages exhibited stronger chemotaxis and cytokine signaling involving innate immune cells (neutrophils, monocytes, and macrophages), whereas adaptive T cell signals were weaker in the AAH/AIS group and stronger in the MIA and IAC groups (Fig. 3c–g). Increased regulatory T cells (Tregs) in IAC samples suggested an immunosuppressive trend during tumor progression (Fig. 3e), potentially regulated by cytokines such as interleukin-6 (IL-6), interleukin-10 (IL-10), and tumor necrosis factor-alpha (TNF-α) (Fig. 3c-g and S3c-f). In the IAC stage, gene expression was enriched mainly in cell proliferation- and division-related signaling pathways (Fig. S3c–e). The dynamic landscape of early-stage LUAD progression highlights invasion as a critical turning point characterized by enhanced transcriptomic heterogeneity and remodeling of the immune microenvironment.

### Tumor progression remodeled the immune microenvironment and dynamic changes in immune cell infiltration

**Fig. 4:**
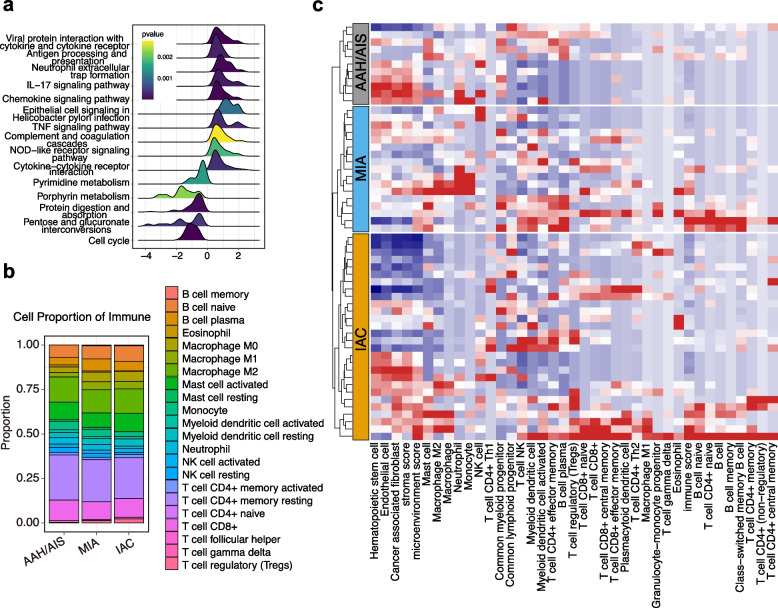
Immune microenvironment characteristics of different subtypes of early-stage LUAD. **a** Gene Set Enrichment Analysis (GSEA) enrichment analysis of differential genes between pre-invasive and invasive LUAD. **b** CIBERSORT-based immune infiltration analysis showed the proportion of immune cell infiltration in the microenvironment of different pathological subtypes. **c** Heatmap of XCell-based immune infiltration analysis of different pathological subtypes

GSEA revealed that during the transition from pre-invasive to invasive LUAD, cell cycle progression and pentose phosphate pathway activity were upregulated, whereas antigen processing and presentation and the IL-17, TNF, and chemokine signaling pathways were markedly downregulated (Fig. 4a and S4a). These results suggest that early tumor progression actively suppresses key immune response pathways to create an immunosuppressive microenvironment.

**Fig. 5:**
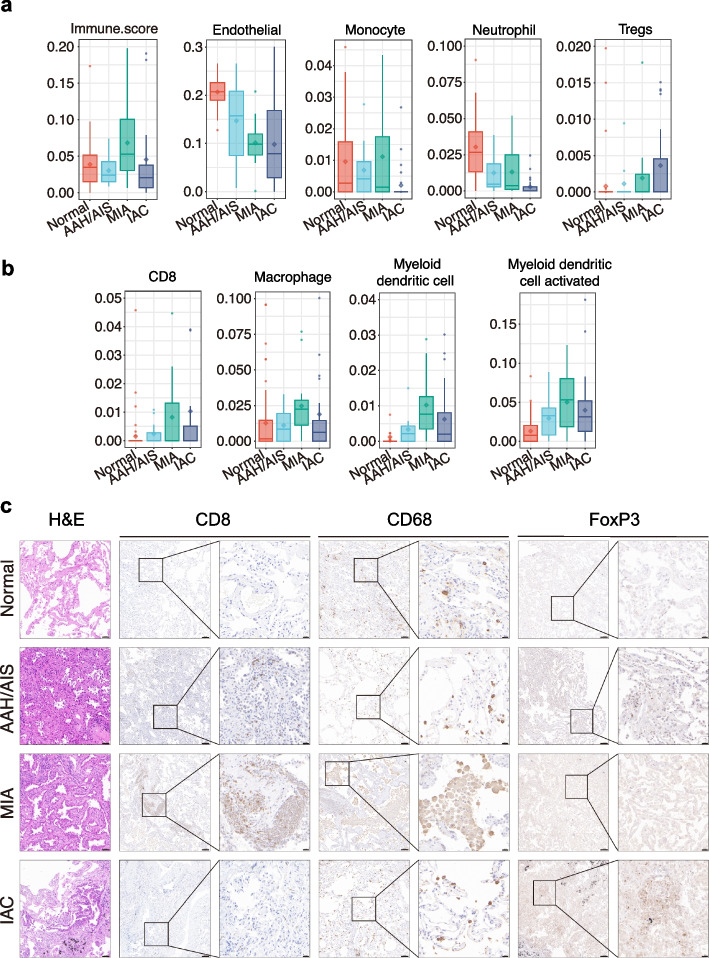
Immune-cell subpopulation infiltration analysis and validation. **a** XCell-based immune scores, along with endothelial cell, monocyte, and neutrophil infiltration, as well as regulatory T cell infiltration, in different pathological subtypes. **b** Infiltration of CD8 + T cells, macrophage, and myeloid dendritic cell (activated) in different pathological subtypes. Data are shown as median ± interquartile range (IQR). **c** Representative hematoxylin and eosin (HE) staining and immunohistochemical (IHC) staining for CD8/CD68/FoxP3 in tumors at different progression stage and matched normal tissues. Scale bar: 50 µm (HE, 200 ×), 100 µm (IHC, 100 ×), 20 µm (IHC, 400 ×)

To further quantify immune cell composition changes, immune infiltration analysis was conducted using CIBERSORT and XCell. The CIBERSORT results illustrated the proportional distribution of different immune cell subtypes (Fig. S4b). The immune cells in the LUAD microenvironment consisted predominantly of B cells, T cells, macrophages, and mast cells, with proportions varying across pathological stages (Fig. 4b). XCell-based immune infiltration scores (Fig. 4c) revealed significant inter-patient heterogeneity, even within the same pathological stage. Overall, innate immunity dominated AAH/AIS, with lower adaptive immune infiltration. Adaptive immune infiltration significantly increased in the MIA stage. In the IAC stage, heterogeneity was evident, with some patients showing MIA-like infiltration patterns and others demonstrating decreased infiltration (Fig. 5a).

Inter-group comparisons of various immune cell subtypes revealed that endothelial cell, monocyte, and neutrophil infiltration decreased with advancing pathology, whereas regulatory T-cell (Treg) infiltration increased (Fig. 5a). Notably, the infiltration of CD8 + T cells, macrophages, and myeloid dendritic cells (mDCs) increased in the early stages (AAH/AIS/MIA) but decreased upon progression to IAC (Fig. 5b). Considering the established roles of Tregs, CD8 + T cells, and macrophages in tumor immunity, IHC staining was performed to evaluate local immune infiltration. FoxP3 (Treg) expression progressively increased throughout tumor progression (normal tissue → AAH/AIS → MIA → IAC). In contrast, CD8 + T cell (CD8) and macrophage (CD68) expression gradually increased from normal lung tissue through AAH/AIS to MIA but significantly decreased with progression to IAC (Fig. 5c).

This trend might reflect an imbalance between tumor progression and the immune response. In the early stages, antitumor immunity is activated, increasing immune infiltration. As the tumor advances, it reshapes the microenvironment to promote immune suppression, consequently causing a reduction in infiltration.

### The negative correlation between early-stage LUAD clinical features and TERT + leukocytes suggested the presence of systemic immune alterations during tumor progression

Based on previous studies, we employed a TERT-based protocol [19–21] to detect TERT-positive immune cells and CTCs in peripheral blood in this study (Fig. S5a-e). To investigate whether immune alterations associated with tumor progression were localized to the primary tumor or reflected systemic changes, peripheral blood from early-stage LUAD patients with different pathological subtypes was pretreated, and the percentage of TERT + leukocytes was analyzed. CTC levels were also examined, given their role in recurrence and metastasis. TERT + leukocyte and CTC counts were measured in 389 patients (clinicopathological characteristics are shown in Table 2). Postoperative pathology reports documented the pathological subtype for each patient: 119 pre-invasive (1 AAH, 41 AIS, 77 MIA) and 270 invasive LUAD cases. The distribution of most clinical characteristics in the prospective cohort was consistent with that of the transcriptome sequencing samples, except for pathological progression type (the grouping criterion).

**Fig. 6:**
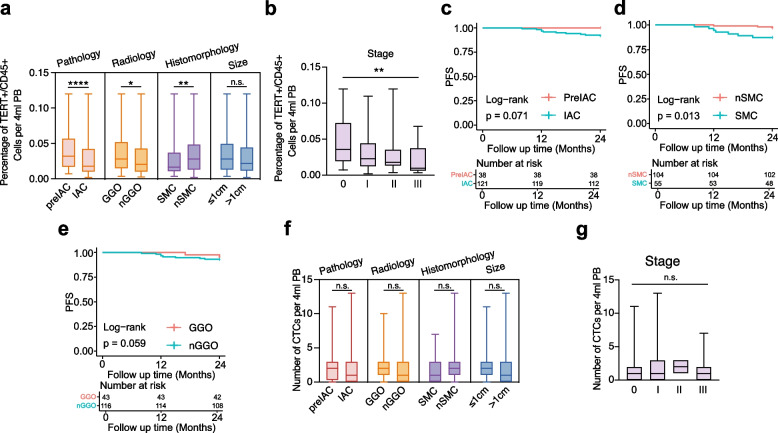
The negative correlation between early-stage LUAD clinical features and TERT + leukocytes suggest systemic immune alterations during tumor progression. **a** Correlation between preoperative baseline telomerase reverse transcriptase (TERT)-positive leukocyte levels and clinical characteristics grouped by pathology, radiology, histomorphology, and tumor size. Data were tested using the non-parametric Mann–Whitney U test. **b** Correlation between preoperative baseline TERT + leukocyte levels and tumor stage. Data were tested using Kruskal -Wallis test. **c-e** Kaplan–Meier curves for PFS in 2-year follow-up patients of prospective cohort stratified according to pathological subtype (**c**), histologic characteristics (**d**), imaging characteristics (**e**). *P*-values were calculated by the log-rank test. **f** Correlation between preoperative baseline CTC levels and clinical characteristics grouped by pathology, radiology, histomorphology, and tumor size. Data were tested using the non-parametric Mann–Whitney U test. **g** Correlation between preoperative baseline circulating tumor cell (CTC) levels and tumor stage. Data were tested using Kruskal -Wallis test. All data were shown as median ± IQR. n.s., *p* > 0.05; *, *p* < 0.05; **, *p* < 0.01; ***, *p* < 0.001; ****, *p* < 0.0001

TERT + leukocyte counts were significantly negatively correlated with the degree of pathological invasion. Compared with patients with invasive LUAD, patients with preinvasive LUAD had significantly greater percentages of TERT + leukocytes in peripheral blood (*p* < 0.0001; Fig. 6a). Similar differences in the TERT + leukocyte percentage were observed between patients with and without SMC (non-SMC; *p* = 0.006) and between patients with GGO and those with nGGO (*p* = 0.01; Fig. 6a). Analysis by clinical stage revealed a significant decreasing trend in the TERT + leukocyte percentage as the stage advanced from 0 to III (*p* = 0.0026; Fig. 6b). Analysis of 159 patients with a 2-year follow-up revealed a negative correlation between PFS and tumor progression, consistent with TERT + leukocyte changes (Fig. 6c–e, Table S1). However, no significant correlation was observed between CTC levels and clinical features (Fig. 6f–g), such as pathological subtype (*p* = 0.68), tumor stage (*p* = 0.43), tumor size (*p* = 0.17), or imaging features (*p* = 0.71).

To assess the potential influence of age distribution on TERT + leukocyte levels, a supplementary analysis was performed. A significant negative correlation was found between the TERT + leukocyte percentage and age: patients ≥ 60 years old had significantly lower levels than those < 60 years old (Fig. S5f). This age-related difference persisted in the invasive LUAD subgroup but was not significant in the pre-invasive subgroup (Fig. S5g). To control for the confounding effect of age, patients were stratified into two age groups (< 60 and ≥ 60 years) and reanalyzed. Within both age strata, patients with invasive LUAD presented significantly lower TERT + leukocyte percentages compared to those with pre-invasive LUAD (Fig. S5h). These findings indicate that although age contributes to TERT + leukocyte levels, tumor progression has a greater effect.

These findings suggest that peripheral blood TERT + leukocytes may serve as potential systemic immune biomarkers reflecting early-stage LUAD progression and prognosis. TERT + leukocyte alterations occurred earlier than CTC detection and were closely associated with tumor invasion depth and patient outcomes.

## Discussion

Lung adenocarcinoma is the leading cause of cancer-related death [22], with clinical outcomes declining sharply with tumor progression and stage [1]. While patients with stage I LUAD exhibit a favorable five-year survival rate of nearly 100%, this rate decreases to less than 7% for those with stage IV LUAD [23]. Adjuvant therapy is generally not recommended for stage 0-I patients after resection, as a subset remains at risk of recurrence and metastasis [24–27]. The retrospective cohort of 601 patients in this study confirms this risk, with stage I patients having a five-year recurrence rate of approximately 10%. Recurrence and metastasis in this cohort occurred exclusively in invasive adenocarcinomas; other clinical features significantly associated with survival stratification (SMC, nGGO, tumor size, and stage) also reflected tumor progression [28]. While progression from atypical adenomatous hyperplasia to invasive adenocarcinoma is generally considered a stepwise process, our results underscore invasion as a critical step with significant biological implications [4, 29, 30]. Our study provides preliminary insights into the accompanying biological changes from both local tumor and systemic perspectives.

The transcriptional expression profile from AAH to IAC reveals local molecular biological changes during early-stage lung adenocarcinoma progression [31]. Although basement membrane disruption occurs in the MIA stage, differential expression analysis revealed that qualitative changes in gene expression are most apparent in the IAC stage, with limited differences between AAH/AIS and MIA. Notably, molecular biological changes observed during invasion primarily involve microenvironmental remodeling rather than changes within the tumor itself.

This remodeling begins with the mobilization of innate immune components, particularly the chemotaxis of innate immune cells such as neutrophils and monocytes, which decreases with advancing disease progression. Concurrently, there is an increase in the infiltration of immunosuppressive Tregs. Adaptive immune responses initially increase in the early stages of tumor progression but then diminish after the IAC stage. These changes reflect a gradual imbalance in the immune microenvironment during tumor education [10–13]. This process, involving a variety of cytokines and immune-related pathways often with bidirectional functions, highlights the delicate balance involved in immune environment remodeling, in which tumor progression ultimately shifts toward an immunosuppressive state.

Consistent with our findings, previous studies based on TCR analysis and immunohistochemistry have reported downregulation of immune activation pathways, upregulation of immune suppression pathways, reduced CTL infiltration, increased Treg infiltration, and a shift toward PD-L1 + CD8 + T cell subtypes (adaptive resistance) as tumors progress from AIS/MIA to IAC [12, 13]. This overall represents a reduction in the immune response and a shift toward an immunosuppressive and potentially immune-failing state.

Our study expands upon these findings, demonstrating that immune microenvironment remodeling extends beyond CTLs to encompass the broader immune response, notably impacting the functions of innate immune components such as dendritic cells and macrophages, which are responsible for immunotransmission. Furthermore, our data indicate that blood vessels and adhesion components within the microenvironment are also reshaped during progression, resulting in a milieu more conducive to tumor invasion and metastasis. The expression profile changes within the tumor itself primarily reflect enhanced cell proliferation.

Conversely, CD45 + /TERT + immune cells were identified in peripheral blood and were significantly negatively correlated with pathological progression. Large cohort studies have demonstrated that telomere shortening in peripheral blood is associated with poor prognosis in cancer patients and adverse health outcomes in the general population [32–34]. This suggests that while the exact biological role of telomerase-active immune cells in peripheral blood remains unclear, they may serve a specific gatekeeping function in cancer and represent a useful indicator for repeated monitoring of systemic immune status [35, 36].

While TERT is not a direct effector molecule in antitumor immunity, it is crucial for maintaining the replicative capacity and long-term protective function of T cells. Specifically, it promotes the formation of memory T cells, which are essential for long-term immune surveillance against tumor recurrence. Many studies have demonstrated a positive correlation between hTERT expression and T cell proliferation and the immune response [15–17]. Haralambieva et al. reported that hTERT expression correlated positively with an increase in specific memory B cells after influenza vaccination [18].

TERT may also function in innate immune cells such as dendritic cells (DCs) and natural killer (NK) cells. Studies suggest that telomerase activity helps maintain DC survival and function, whereas the sustained activity and expansion of NK cells may benefit from TERT-mediated telomere maintenance. Therefore, TERT + leukocytes may represent the long-term functional reserve of multiple effector cell populations in antitumor immunity, offering significant research value for predicting survival in tumor patients. A reduction in TERT + cells can serve as a quantitative and measurable indicator of the shift from “immune control of the tumor” to “tumor suppression of immunity”. However, the specific mechanisms through which hTERT expression in immune cells directly correlates with antitumor immunocompetence remain unexplored. Our study takes the first crucial step toward addressing this gap by demonstrating that the proportion of TERT + leukocytes decreases significantly with tumor progression. This reflects a decline in a functional and replication-competent immune cell reservoir, indicating that the systemic immune status of tumor patients is affected by the primary tumor and is skewed toward a state of hypoimmunoreactivity as the tumor progresses.

While preliminary studies have revealed remodeling of both local and peripheral immunity toward immunosuppression in early-stage LUAD, the dynamic relationship between the tumor immune microenvironment (TME) and systemic immune status warrants further investigation. Immune cells within the TME, such as Tregs that secrete inhibitory cytokines, can influence the systemic immune status. The dynamics of TERT + immune cells, which are components of the TME, may reflect the tumor’s impact on overall immunity. Understanding this local-systemic immune interplay could identify targets for early diagnosis or intervention. TERT + leukocytes hold promise as multipurpose biomarkers for optimizing clinical patient management. Baseline TERT + leukocyte levels may reflect the functional reserve of the immune system, aiding in guiding personalized monitoring and treatment strategies. Dynamic monitoring of their levels during treatment may allow early assessment of immune activation, potentially predicting response or resistance ahead of radiographic changes [37]. Interventions aimed at reversing the loss of functional TERT + immune cells could represent a novel strategy for restoring antitumor immunity.

However, no correlation was observed between CTC levels and clinical characteristics in early-stage LUAD patients. Although previous studies in intermediate-advanced cancers (e.g., hepatocellular carcinoma and pancreatic cancer) reported significant associations between CTCs and TNM stage [38–42], these findings may not apply to early-stage disease. Studies specific to early-stage LUAD also found CTC count correlated only with age and was not significantly related to sex, tumor size, or driver-gene status [43, 44]. Combined with our results, these findings suggest that changes in CTC biological behavior may lag behind pathological progression in early-stage LUAD. Future studies could employ single-cell sorting to isolate CTCs from different disease stages for further biological characterization.

This study has several limitations. First, the exploratory nature of delineating early local biological evolution in LUAD led to a focus on transcriptomic profiling. This precludes detailed cellular subpopulation data and lacks complementary protein-level or metabolic information. Future work will build upon these findings by focusing on immune alterations during early tumor progression and incorporating more comprehensive multi-omic analyses. Second, transcriptional profiling revealed significant heterogeneity among patients at all pathological stages, especially among IAC patients, leading to greater within-group variability. This suggests ongoing tumor progression even after the IAC stage, highlighting the need for patient stratification based on both tumor primary foci and systemic immune status to enable personalized treatment. We acknowledge that the small sample size may introduce bias and affect the generalizability of our findings and the robustness of our conclusions. Third, while the percentage of TERT + immune cells in peripheral blood reflects systemic immune status during tumor progression, and our previous study indicated its significance for suggesting postoperative recurrence in lung adenocarcinoma patients [37], the biological properties of this cell population require further investigation to define the specific immune cell subpopulations they represent..

The clinical translation of TERT + immune cells is currently limited by the complexity of the technical workflow. To address these limitations, future efforts will focus on the following directions. First, a simplified and automated detection process, for example, exploring ready-to-use reporter viral particles or nonviral delivery systems and integrating the entire workflow into an automated platform, should be developed. Second, identifying mRNA or protein markers highly correlated with TERT activity that can serve as alternatives would allow direct detection without transfection. Third, liquid-phase assays based on specific secretory factors (e.g., cytokines and exosomes) from TERT + leukocytes should be developed, enabling noncellular, high-throughput detection. Through these strategies, we aim to establish a standardized, automated, and robust assay to facilitate the broad application of TERT + leukocytes.

## Conclusion

This study provides comprehensive depiction of key biological alterations during the progression of early-stage lung adenocarcinoma from both peripheral and primary tumor foci. Our findings suggest a model in which tumor progression remodels the local and peripheral immune environment toward immunosuppression before significant changes in the tumor’s intrinsic biological behavior occur, lowering the overall immune response and facilitating continued tumor advancement. This highlights the importance of immune-targeted interventions in early-stage LUAD, providing a reliable direction for future research aimed at halting tumor progression.

## Materials and methods

### Study design

A retrospective cohort of patients with stage I LUAD who underwent surgical resection at the Department of Thoracic Surgery, Cancer Hospital, Chinese Academy of Medical Sciences between 2017 and 2019. Inclusion criteria were: (1) written informed consent; (2) no neoadjuvant therapy before surgery; (3) proposed surgical resection; (4) postoperative pathological staging confirming stage I disease; and (5) an Eastern Cooperative Oncology Group (ECOG) performance status of 0 or 1. Exclusion criteria were severe cardiac dysfunction, defined as a left ventricular ejection fraction (LVEF) < 50% or congestive heart failure (CHF) ≥ grade 2 (CTCAE v5.0 or New York Heart Association classification ≥ grade 2). A total of 601 patients were included, and their five-year survival outcomes were assessed.

The prospective cohort included 389 patients with LUAD who underwent surgical resection at the same department between 2021 and 2024. As part of the study protocol, tumor and matched normal tissues were collected intraoperatively, immediately immersed in RNAlater (ThermoFisher), and stored at –80 °C. Following pathological confirmation, samples were randomly selected for transcriptome sequencing. Preoperative peripheral blood (4 mL) was collected in EDTA anticoagulant tubes and transported to the laboratory for TERT + leukocyte and CTC analysis. A flowchart illustrating the prospective cohort study design is shown in Fig. 1.

### Transcriptome sequencing and analysis

Tumor and matched adjacent normal tissues representing early-stage LUAD (AAH to IAC) were selected for transcriptome sequencing. Given a clinical surgical ratio of pre-IAC to IAC tumors of approximately 1:2, a 1:1 ratio was maintained in the sequencing analysis to mitigate analytical bias due to unequal group sizes. Samples within each group were randomly selected from the prospective cohort based on pathological subtype. Total RNA extracted from tissue samples was assessed for RNA integrity number (RIN) score and concentration utilizing the Agilent 2100 RNA Nano 6000 Assay Kit (Agilent Technologies). Qualified samples were used for library preparation with the VAHTS Universal V10 RNA-seq Library Prep Kit (Vazyme) and quality was assessed with Qubit3.0 and Agilent 2100. Libraries passing quality control were sequenced on the NovaSeq 6000 platform (PE150 sequencing strategy).

Raw sequencing data were filtered and aligned to generate an expression matrix. Differential gene expression analysis was conducted using DESeq2, with differential genes defined as those exhibiting a fold change ≥ 1.5, a *p*-value ≤ 0.05, and a Benjamini–Hochberg adjusted p-value (P.adj) ≤ 0.05. Gene Ontology (GO), Kyoto Encyclopedia of Genes and Genomes (KEGG), and Gene Set Enrichment Analysis (GSEA) pathway enrichment analyses were performed using ClusterProfiler. Immune cell infiltration was inferred from transcriptome data using CIBERSORT and XCell.

### Blood sampling and TERT + leukocytes/CTC detection

The mechanism and protocol for TERT-based detection of CTCs and TERT+ leukocytes are detailed in Fig. S1. Peripheral blood samples (4 mL) were collected in EDTA anticoagulant tubes and centrifuged to remove plasma. The remaining blood cells were lysed and resuspended in serum-free medium. The cell suspension was transfected with a herpes simplex virus type 1 (HSV1) vector encoding green fluorescent protein (GFP) under the control of the human telomerase reverse transcriptase promoter (hTERTp) at a multiplicity of infection (MOI) of 1. After 24 h of incubation, cells were collected and stained with anti-CD45 antibodies (eBioscience™, APC, clone: HI30). TERT + leukocytes were identified as CD45 + /GFP- cells, and CTCs were identified as CD45-/GFP + cells via flow cytometry.

### Histology and immunohistochemical staining

Tumor and matched normal tissues from each tumor-invasion stage were paraffin-embedded and sectioned according to standard protocols. Paraffin sections were subjected to hematoxylin and eosin (H&E) staining and validated by immunohistochemical (IHC) staining. The following primary antibodies were used: CD8 (Abcam, clone: CAL66), CD68 (Abcam, clone: EPR20545), and FoxP3 (Abcam, clone: 236A/E7).

### Statistical analyses

Statistical analyses were performed using R software (version 4.1.2). The association between clinical characteristics and survival outcomes was assessed using Kaplan–Meier analysis. Nonparametric Mann–Whitney U tests and Kruskal–Wallis tests were used to compare TERT + leukocyte and CTC levels (continuous, non-normally distributed data). All *p*-values were two-sided, and *p* < 0.05 was considered statistically significant. *p*-value adjustments were implemented with Bonferroni correction. This approach is a conservative statistical technique that helps control the family-wise error rate, ensuring the reliability of findings.

## Supplementary Information

Supplementary Material 1.

## Data Availability

Transcriptomic data supporting this study's findings are available from the corresponding author upon reasonable request. No new algorithms were developed for this study. All codes generated for analysis are available.
