# Supplementary material for: Peripheral TERT- positive leukocytes as a biomarker of the local and systemic immune failure in early-stage lung adenocarcinoma progression
Source: Mol Biomed. 2025 Dec 19;6:146. doi: 10.1186/s43556-025-00383-3 (PMC12717345; doi:10.1186/s43556-025-00383-3)
Supplement: Supplementary file 1 — Supplementary Material 1. [file 43556_2025_383_MOESM1_ESM.pdf]

Peripheral TERT- positive leukocytes as a biomarker of the local and systemic immune failure in early-stage lung adenocarcinoma progression

Qi Zhang[1, #], Xiaoli Zhang[2, #], Guoliang Li[3], Ligong Yuan[4], Duo Wan[2], Peipei Xie[2], Shujun Cheng[2], Yu Zhang[5], Kaitai Zhang[2, *], Yousheng Mao[6, *], Wen Zhang[5, *]

[1]Department of Gastroenterology, Beijing Friendship Hospital, Capital Medical University, State Key Laboratory for Digestive Health, National Clinical Research Center for Digestive Diseases, Beijing, China.

[2]State Key Laboratory of Molecular Oncology, Department of Etiology and Carcinogenesis, National Cancer Center/National Clinical Research Center for Cancer/Cancer Hospital, Chinese Academy of Medical Sciences and Peking Union Medical College, Beijing, China.

[3]Department of Radiation Oncology, The Affiliated Hospital of Qingdao University, Qingdao, China.

[4]Department of Thoracic Surgery, the First Affiliated Hospital of USTC, Division of Life Sciences and Medicine, University of Science and Technology of China, Hefei, 230001, China.

[5]Department of Immunology, National Cancer Center/National Clinical Research Center for Cancer/Cancer Hospital, Chinese Academy of Medical Sciences and Peking Union Medical College, Beijing 100021, China.

[6]Department of Thoracic Surgery, National Cancer Center/National Clinical Research Center for Cancer/Cancer Hospital, Chinese Academy of Medical Sciences and Peking Union Medical College, Beijing 100021, China.

[#]These authors contributed equally: Qi Zhang, Xiaoli Zhang

Supplementary Information

Supplementary Table

Supplementary Figures

Supplementary Figure legends

Table S1 Clinical and pathological characteristics of patients who have completed 2 years of follow-up in the prospective cohort.

Characteristics	All patients(N=159)
Tumor size (average, cm)	1.79
Neurological violation, n (%)	6(3.8)
Vessel carcinoma embolus, n (%)	15(9.4)
Median age (years, range)	60(34-82)
Gender, n (%)	
Male	62(39.0)
Female	97(61.0)
Smoking history, n (%)	
Smoker	41(25.8)
Nonsmoker	118(74.2)
Clinical TNM stage, n (%)	
0-I	137(86.2)
II	8(5)
III	14(8.8)
Pathology, n (%)	
AIS	10(6.3)
MIA	28(17.6)
IAC	121(76.1)
Adjuvant therapy, n (%)	
Chemotherapy	12(7.5)
Targeted therapy	6(3.7)
Chemotherapy + Immunotherapy	6(3.7)
Chemotherapy +Targeted therapy	6(3.7)
Radiological features, n (%)	
Solid	60(36.5)
Sub-Solid	60(37.7)
GGO	39(37.7)
Surgical procedure, n (%)	
Lobectomy	93(58.5)
Sublobar resection	66(41.5)
Histomorphology, n (%)	
SMC	53(33.3)
nSMC	106(66.7)
Tumor location, n (%)	
LUL	46(28.9)
LLL	24(15.1)
RUL	57(35.9)
RML	5(3.1)
RLL	27(17.0)
Recurrence, n (%)	
Intrapulmonary	8(5.0)

Brain	1(0.6)
Lung + Lymph mode	1(0.6)
No recurrence	149(93.8)

n(%) :the number of patients within each category(corresponding percentage of that category); AIS: adenocarcinoma in situ; MIA: minimally invasive adenocarcinoma; IAC: invasive subtype of lung adenocarcinoma; GGO: ground glass opacity; SMC: solid and micropapillary components; nSMC: non-solid and micropapillary components; LUL: left upper lobe; LLL: left lower lobe; RUL: right upper lobe; RML: right middle lobe; RLL: right lower lobe.

Supplementary Figure 1

a

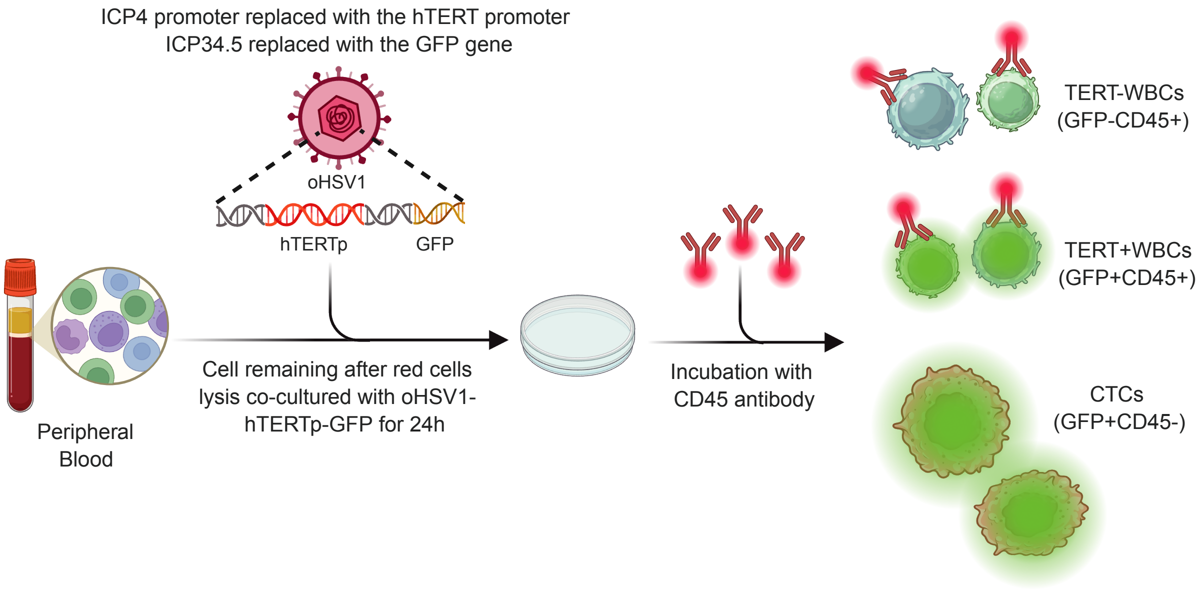

Supplementary Figure 2

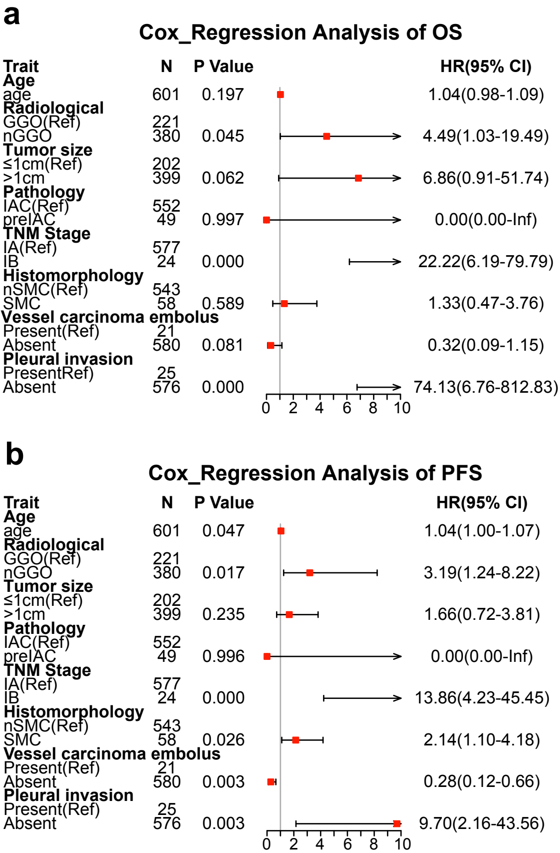

Supplementary Figure 3

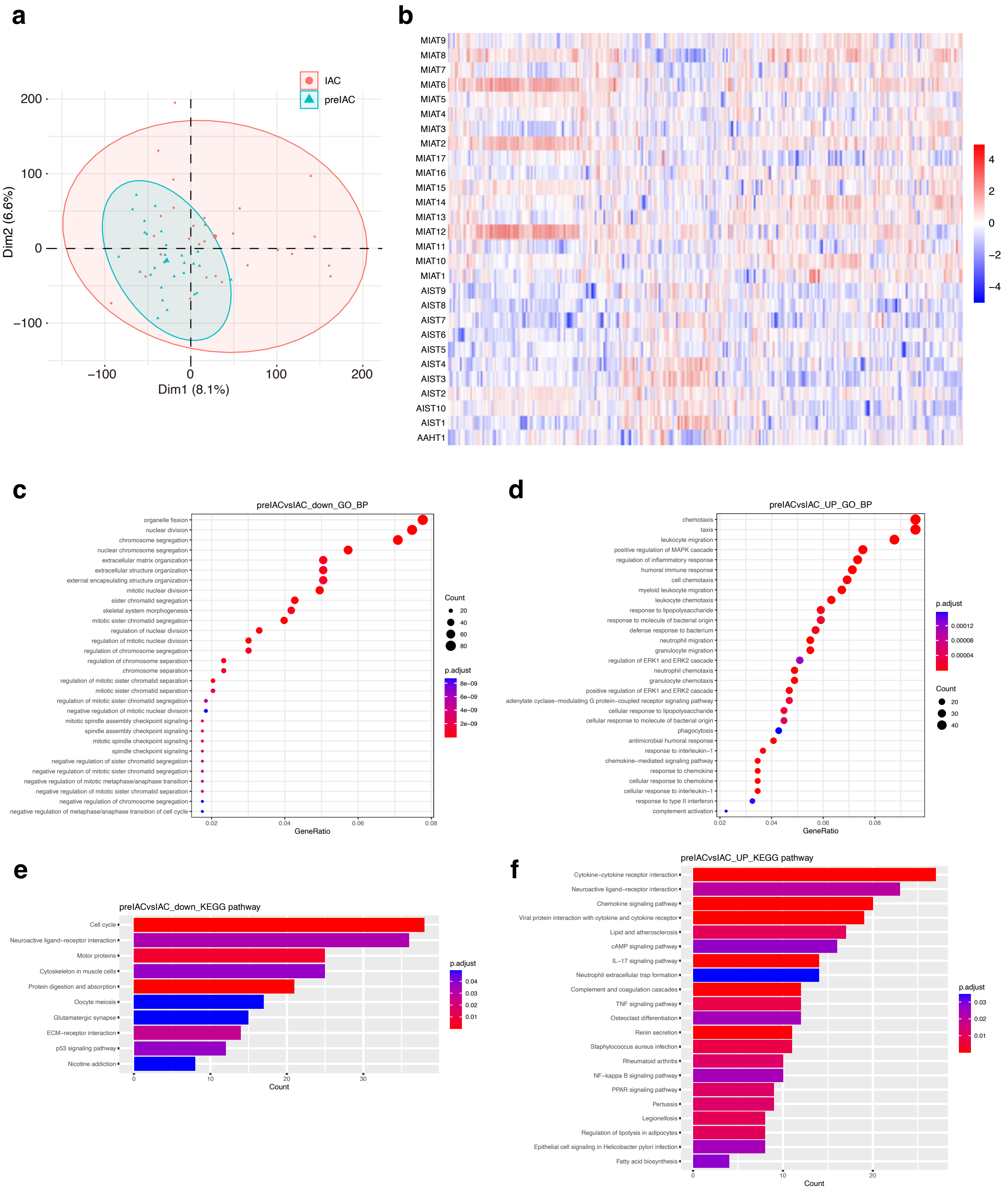

Supplementary Figure 4

a

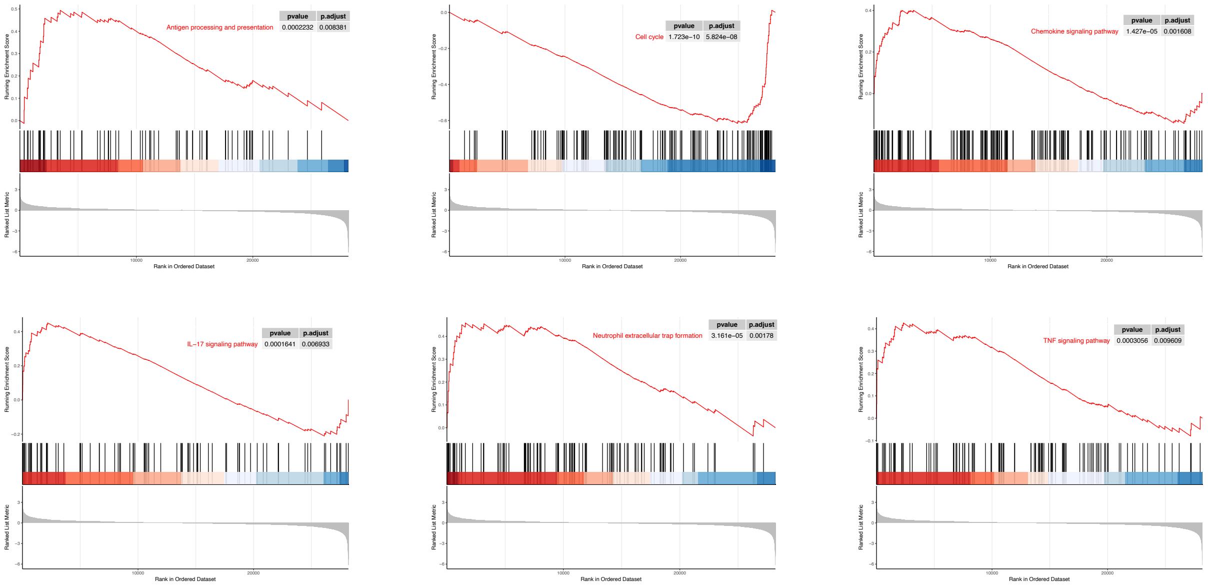

b

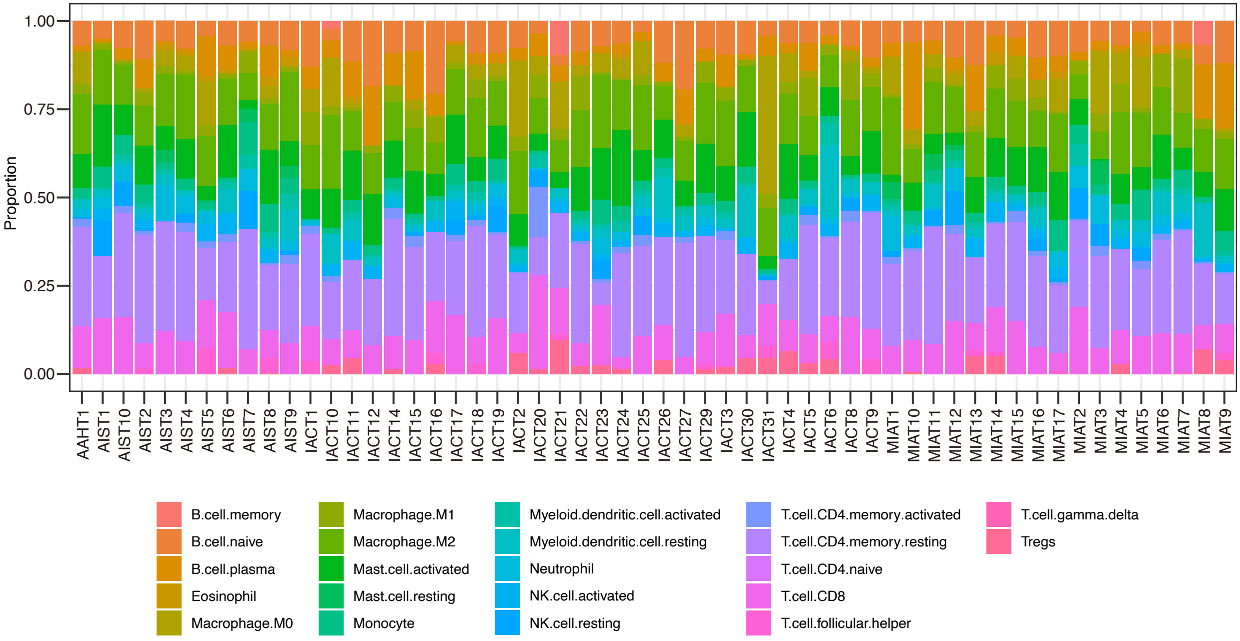

Supplementary Figure 5

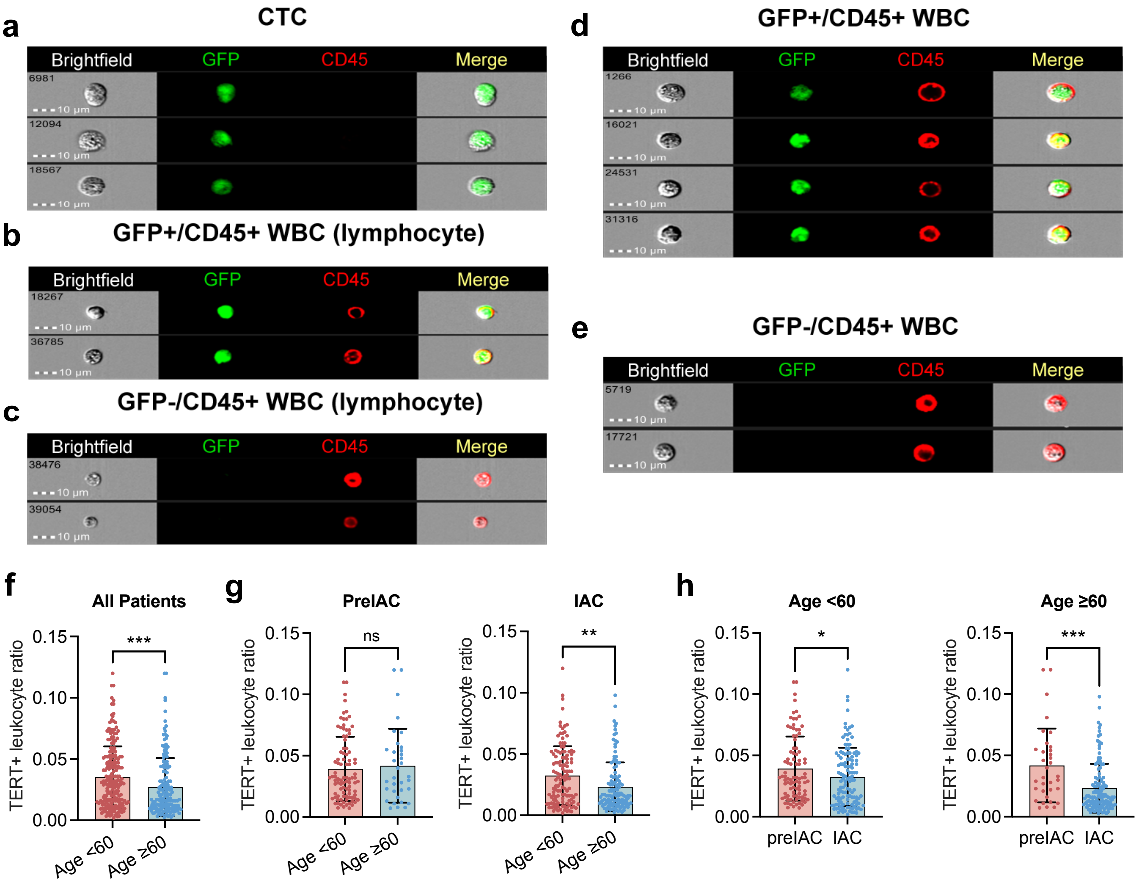

Supplementary Figure legends

Figure S1 Mechanistic diagram of the TERT-based detection protocol for CTCs and TERT+ leukocytes.

Figure S2 Cox regression analysis of recurrence and metastasis characteristics in patients with stage I LUAD. (a) Forest plot of univariate Cox regression of OS for clinicopathologic variables. **(b)** Forest plot of univariate Cox regression of PFS for clinicopathologic variables.

Figure S3 Transcriptome landscape from pre-invasive lung adenocarcinoma to invasive lung adenocarcinoma. (a) PCA plot for pre-invasive and invasive lung adenocarcinoma. **(b)** Heatmap of differential gene analysis between AAH/AIS and MIA. **(c-d)** GO enrichment analysis of down-(c) and up-(d)regulated differential genes in pre-IAC versus IAC. **(e-f)** KEGG enrichment analysis of down- (e) and up- (f) regulated differential genes in pre-IAC versus IAC.

Figure S4 GSEA enriched pathways and single sample immune cell infiltration percentages. (a) Representative GSEA-enriched pathways for differential genes in pre-IAC versus IAC. **(b)** Percentage of immune cell infiltration in each individual sample.

Figure S5 Representative FlowSight images of CTCs/ WBCs and correlation analysis between TERT+ leukocytes and age. (a) CTC captured by flow imaging (CD45-/GFP+). **(b-c)** TERT+(GFP+) or TERT-(GFP-) lymphocytes captured by flow imaging (CD45+). **(d-e)** TERT+(GFP+) or TERT-(GFP-) leukocytes captured by flow imaging (CD45+). Scale bar: 10 μm. **(f)** Quantification of the TERT+ leukocyte ratio across all patients. **(g)** Subgroup analysis of the TERT+ leukocyte ratio based on age. **(h)** Subgroup analysis of the TERT+ leukocyte ratio based on pathology. Each dot represents a single patient, and data are presented as mean with standard deviation (SD). An uEnpaired Student's t-test was used for statistical comparisons.

ns, not significant; $p < 0.05$; $*p < 0.01$; $**p < 0.001$.
